# The use of diet for preventing and treating depression in young men: current evidence and existing challenges

**DOI:** 10.1017/S000711452300168X

**Published:** 2024-01-28

**Authors:** Jessica Bayes, Janet Schloss, David Sibbritt

**Affiliations:** 1 National Centre for Naturopathic Medicine, Southern Cross University, Lismore, NSW 2480, Australia; 2 School of Public Health, University of Technology Sydney, Ultimo, NSW 2007, Australia

**Keywords:** Nutritional psychiatry, Young adults, Men, Mediterranean Diet

## Abstract

Emerging evidence suggests that diet therapy (nutrients, foods and dietary patterns) could be effective as a potential adjunctive treatment option for major depressive disorder. Numerous mechanisms have been proposed, including the role inflammation, oxidative stress, brain-derived neurotrophic factor, the gastrointestinal tract microbiome and tryptophan/serotonin metabolism. Despite known differences in depression characteristics and treatment responses between males and females, there are limited sex-specific studies examining the role of diet in young men specifically. This is important as young men are often reluctant to seek mental health support, so finding treatment strategies which appeal to this demographic is crucial. This brief report provides an overview of the most recent advances in the use of diet for preventing and treating depression in young men, highlighting existing challenges and opportunities for future research. We recommend that clinicians discuss the role of diet with depressed young men, so that diet may be used alongside current treatment options.

## Graphical abstract

**Figure:**
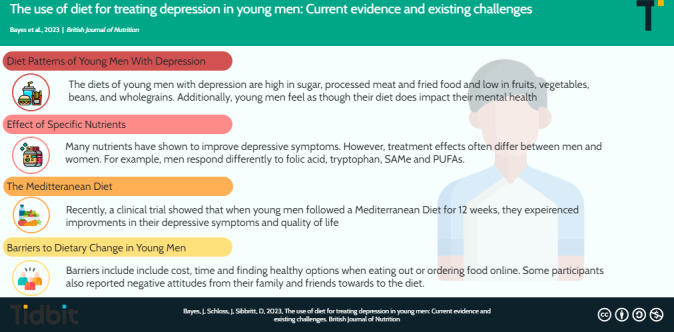

## Short communications

Depression is a leading cause of disability among young adults, and in Australia, depression affects 6 % of 18–25 year olds each year^(1)^. Many young adults cannot correctly identify the symptoms of depression^(2)^ and have limited understanding about effective treatment options^(3)^. Research shows that men are less likely to seek help than women, with only one in four men who experience depression accessing treatment^(4)^. Depression is also a high-risk factor for suicide, which is the leading cause of death in young men^(5)^. Therefore, there is a pressing need for early interventions which appeal to young men with depression. Recently, diet therapy, which encompasses individual nutrients, foods and dietary patterns, has been proposed as a potential adjunctive treatment option for major depressive disorder.

Nutritional psychiatry explores the use of specific nutrients, foods and dietary patterns for mental health conditions^(6)^. A large number of different nutrients have been examined for their effect on depressive symptoms, including vitamins such as folate, B_12_ and B_6_^(7)^, amino acids such as tryptophan^(8)^ and S-adenosyl-L-methionine (SAMe)^(9)^, as well as polyphenols^(10)^, probiotics^(11)^ and *n*-3 fatty acids^(12)^. Numerous mechanisms whereby diet may exert beneficial effects on depressive symptoms have been proposed. These include the role of reducing inflammation, oxidative stress, increasing brain-derived neurotrophic factor, balancing the gastrointestinal tract microbiome and tryptophan/serotonin metabolism^(13)^. Despite known differences in depression characteristics and treatment responses between males and females, there are limited sex-specific studies examining the role of diet in young men specifically.

### Specific nutrients, diets and depression in men: tryptophan, S-adenosyl-L-methionine, folic acid, polyphenols and *n*-3 fatty acids

Tryptophan is an essential amino acid that acts as a precursor to serotonin^(14)^. The role of tryptophan in mood disorders has been the target of much research since the 1980s; however, the results are often inconsistent and contradictory^(15)^. Further research suggests that men and women respond differently to increases in tryptophan levels^(15)^, with tryptophan supplementation found to affect the emotional processing response of females, but not males^(16)^. This indicates that women may be more sensitive to changes in serotonin levels than men^(16)^ with studies showing that oestrogen and testosterone exert direct and indirect effects on serotonin transporter proteins^(17)^. In addition, a large epidemiological study of 29 133 Finnish men found no association between dietary tryptophan intake and self-reported depressed mood, hospital admission for depressive disorders or suicide^(18)^. The conflicting evidence on the role of tryptophan in men currently limits recommendations for its use at this time.

SAMe is a nutritional compound that plays a crucial role in the one-carbon cycle and methylation of neurotransmitters^(19)^. Several studies have assessed the effect of SAMe supplements for treating mood disorders, with the majority showing a favourable effect^(19)^. However, research demonstrates that sex might impact the antidepressant efficacy of SAMe, with a greater therapeutic effect observed in men^(20)^. This finding emerged despite both males and females displaying similar baseline depression severity. The authors suggest this finding could be due to variances in the one-carbon cycle pathways, as a result of hormonal regulation^(20)^. However, further research is needed in this area to determine the exact mechanisms involved.

Folic acid also plays an important role in the one-carbon cycle, and folate deficiency has consistently been associated with increased depression^(21)^. A recent systematic literature review and meta-analysis of five randomised control trials found that folate, as an adjunct to selective serotonin reuptake inhibitors and serotonin and norepinephrine reuptake inhibitors, improves depression scores, remission and response rates^(21)^. However, sex differences have been observed, with a 10 week trial demonstrating that a significantly greater percentage of women responded favourably to the folic acid supplementation compared with the men^(22)^. It has been suggested that the 500-µg/d dose may not have been sufficient for men and that a higher does may be required to achieve a positive treatment effect^(23)^.

Other nutritional compounds that have received recent attention for their potential impact on depression are the various polyphenolic compounds^(10)^. Polyphenols are natural agents found in a variety of plant foods^(24)^. These naturally occurring compounds are found in high quantities in fruits, vegetables, tea, coffee, chocolate, legumes and cereals^(24)^. Polyphenols are potent antioxidant and anti-inflammatory agents and have shown to prevent neuro-inflammation^(25)^ and modulate specific cellular signalling pathways involved in cognitive processes^(26)^. A recent systematic literature review of seventeen experimental and twenty observational studies assessed the role of polyphenols for depression^(10)^. The results highlighted a beneficial role of several different polyphenols in reducing depression risk and symptoms, including coffee, curcumin, soy isoflavones, tea and cocoa flavanols, walnut flavonols, citrus flavanones and the stilbene resveratrol^(10)^. However, the review also highlighted a lack of research on men and young adults specifically^(10)^.

Another nutrient which has gained much recent attention is *n*-3 PUFA. While much evidence supports the efficacy of *n*-3’s for depression^(12)^, numerous epidemiological studies have found these beneficial effects apply to females but not to males^(27–29)^. A review of fifteen epidemiological studies has suggested that these differences could possibly be due to sex hormones and their influence on DHA^(30)^. Oestrogen has shown to increase DHA levels within the body, while testosterone decreases them^(31)^. This further demonstrates the different treatment response experienced by men to certain nutrients.

A recent systematic review and meta-analysis published in 2019 assessed the effect of dietary improvement on the symptoms of depression and anxiety^(32)^. They found that studies with female samples observed significantly greater benefits from dietary interventions compared with those with male samples^(32)^. The authors propose three reasons to explain this finding. First, that females have higher rates of mood disorders across the general population^(32)^; however, this may be because men under report the condition^(33)^. Second, due to differences in metabolism and body composition, females may be more responsive to dietary interventions which affect glucose or fat metabolism^(32)^. Third, the sociocultural differences between males and females may shape beliefs around diet, nutrition and health thereby influencing the outcome of dietary intervention trials^(32)^.

### Dietary patterns of young men with depression

Currently, there has been a shift away from supplementing with isolated nutritional compounds to a focus on overall dietary patterns^(34)^. This shift is due to the recognition that individuals do not consume single nutrients, rather, nutrients are consumed in whole-of-diet patterns^(34)^. A recent Australian cross-sectional study, *the MEN’S Diet and Depression Survey e*xamined the diets of 384 young men aged 18–25 years with clinical depression^(35)^. The results revealed that their diets were poor and included a high consumption of processed foods and high sugar snacks^(35)^. The majority of the participants consumed chocolate and sweets three times per week, in addition to a high consumption of sweet pastries and bakery items^(35)^. Research suggests that high sugar intake is associated with low mood due to its influence on inflammation, insulin response and brain-derived neurotrophic factor^(36)^.

There was also a high consumption of fried foods, with the majority of participants consuming pizza, and fried potato such as French fries or hash browns three or more times per week^(35)^. These foods are high in processed vegetable oil which has recently been shown to increase the risk of developing depression^(37)^. Vegetable oil is high in *n*-6 fatty acids, and it is thought that an imbalance in the ratio of *n*-6 and *n*-3 fatty acids contributes to increased inflammation and negative mental health symptoms^(37)^.

Additionally, roughly 40 % of participants in the *MEN’S Diet and Depression Survey* consumed processed meats such as bacon, hot dogs and lunch meats such as ham, salami and spam two or more times per week^(35)^. This is particularly concerning as much epidemiological data has shown that a high intake of processed meat is associated with CVD, type 2 diabetes mellitus and cancer^(38)^. A recent meta-analysis of eight observational studies has also demonstrated that processed meat is associated with a moderately higher risk of depression^(38)^. Processed meats are high in inflammatory compounds such as saturated fats, nitrates and nitrites, polycyclic aromatic hydrocarbons and heterocyclic amines which could be responsible for these negative effects^(39)^.

Interestingly, the *MEN’S Diet and Depression Survey* study also found that the vast majority of the participants indicated that they feel as though their diets impact their mental health and would be willing to change it in order to help their depressive symptoms^(35)^. The results showed that their current diets were low in fruits, vegetables, beans and wholegrains. Only 0·8 % of participants consumed vegetables four or more per day and roughly half of the participants reported never consuming wholegrains or legumes^(35)^. This is significantly less than consumption in women, with a recent longitudinal analysis of Australian women’s fruit and vegetable consumption finding that 3–4 % of women were eating the recommended intake of vegetables per day^(40)^. These foods are staple ingredients in the Mediterranean Diet, which is currently the dietary pattern with the most evidence for treating major depressive disorder^(41,42)^.

### The use of a Mediterranean Diet for treating depression in young men

A recent randomised controlled trial, *A Mediterranean diet for MEN with Depression* (The AMMEND trial) tested the effect of a Mediterranean Diet on the symptoms of depression in young Australian men aged 18–25 years, with moderate to severe clinical depression^(43)^. This 12-week trial involved three appointments with a nutritionist who provided detailed instructions on the diet intervention. The results demonstrated that young men who experience depression are able to significantly change their diet quality over a short time period (12 weeks) under the guidance of a clinical nutritionist^(43)^. The Mediterranean Diet intervention in this cohort led to both clinically and statistically significant improvements in depressive symptoms, as measured by a 20·6 point reduction on the Beck Depression Inventory (*P* = 0·001). Improvements were also observed on the WHO, quality of life measurement scale, with both clinically and statistically significant improvements observed in both the psychological (29 point increase, *P* = 0·001) and physical domain (24 point increase, *P* = 0·001), as well the total quality of life raw score (18 point increase, *P* = 0·001)^(43)^.

These results are in agreement with another study that explored the effect of a brief dietary intervention conducted in young adults^(44)^. This study included both male and female participants aged 17–35 years who displayed elevated symptoms of depression^(44)^. The diet was based on the Australian Guide to Healthy Eating, with additional Mediterranean-style diet components. The diet intervention resulted in significantly lower self-reported depression symptoms^(44)^. Together, these findings suggest that following a Mediterranean Diet has the potential to have a wide impact on young men with depression, influencing many aspects of their health and well-being. Additionally, the Mediterranean Diet was well tolerated in both of these studies with no reported side effects^(43,44)^.

### Key challenges of incorporating dietary change in young men

The AMMEND end of project evaluation identified several barriers experienced by the study participants when following the Mediterranean Diet program^(45)^. These included the cost of Mediterranean Diet foods, time spent cooking and preparing food and struggling to find options which met the diet criteria when eating in restaurants or ordering food online^(45)^. Some participants also reported negative attitudes from their family and friends towards to the diet^(45)^. Despite these challenges, excellent adherence to the diet was observed among all participants, suggesting that the motivation to adhere to the diet was greater than the influence of these barriers^(45)^. However, it is unclear what long-term effect that these challenges would have on ongoing adherence to a Mediterranean Diet.

Previous research which examined a Mediterranean Diet intervention in healthy older adults assessed the facilitators and barriers for adopting a Mediterranean Diet in a non-Mediterranean country^(46)^. They reported similar challenges, including the increased cost of food and time required to prepare food as key barriers affecting their experience. The authors recommended that the assumptions about what a Mediterranean Diet involves should be challenged, as many participants held the view that the Mediterranean Diet predominantly consists of salads^(46)^.

In addition to these barriers, research shows that everyday items, including foods, are permeated with subtle yet pervasive gender associations^(47)^. Studies have demonstrated that individuals who eat unhealthy food and larger portion sizes are typically seen as more masculine^(48,49)^. Conversely, individuals who eat healthy food and smaller meals are perceived as more feminine^(48)^. The food with the strongest association with the masculine identity is meat, with fruits and vegetables frequently viewed as more feminine^(48)^. The impact of gender stereotypes and the desire for social acceptance and the influence that this may have on food choices should not be underestimated^(47)^. It is important that these factors are taken into consideration when evaluating the barriers to implementing a Mediterranean Diet in young men with depression.

### Suggestions for overcoming these challenges in research trials and within clinical practise

When discussing the Mediterranean Diet in clinical practice and in clinical trials, adequately explaining the components of the Mediterranean Diet is crucial in order to correct assumptions about what a Mediterranean Diet involves. Emphasis should also be given to providing low-cost food items and simple recipes which take minimal time to prepare. Providing examples on how to choose healthier options when ordering food online may also be valuable for young men. Discussing strategies to overcome the potential negative attitudes of their family and peers should also be considered.

Some authors have suggested that the word ‘diet’ comes with many negative associations, especially for individuals who have previously experienced restrictive dietary regimens^(50)^. Previous research has also demonstrated that men view dieting as a predominantly female activity^(51)^. Therefore, it is to be expected that a Mediterranean Diet intervention may create conflict with both food-based gender stereotypes and help seeking behaviour in general. It has been suggested that to overcome these negative connotations, the Mediterranean Diet be referred to as ‘a lifestyle’ rather than ‘a diet’^(52)^. It would be interesting to look at what difference, if any, omitting the word ‘diet’ makes to the attitudes of young men. For example, would a ‘Mediterranean Lifestyle’ or ‘Mediterranean Eating Pattern’ be better perceived and understood by this demographic? Determining what words, terms and phrases resonate the most with this demographic may help with clinical trial recruitment and for clinicians suggesting a Mediterranean Diet in clinical practice.

### Conclusion

When considering isolated nutrient supplementation for men, the current evidence is conflicting. The role of tryptophan on depressive symptoms in men is contradictory, and *n*-3 fatty acids appear to have a less pronounced treatment effect in males compared with females. Polyphenols show promise, but more research is required to confirm these results. The shift in nutrition science from isolated nutrients to overall dietary patterns may hold more potential. New evidence shows that a Mediterranean Diet is effective as an adjunctive treatment in reducing symptoms of depression in young men with moderate to severe clinical depression. We recommend that clinicians concentrate on discussing the role of diet with depressed young men and consider referrals to nutritionists or dietitians for specialist support, so that diet may be used alongside current treatment options. Further research investigating other dietary interventions in this population group is also required.
